# Targeting IL-1R1 and androgen receptor using natural compounds from *Aconitum heterophyllum* and *Podophyllum hexandrum*: an *in silico* and *in vitro* study

**DOI:** 10.3389/fphar.2026.1909943

**Published:** 2026-10-27

**Authors:** Mir Muskan Un Nisa, Parvez Ahmad Sofi, Peerzada Ishtiyak Ahmad, Rashid Mir, Talib Bashir Bhat

**Affiliations:** 1 Division of Forest Products and Utilization, Faculty of Forestry, Sher-E-Kashmir University of Agricultural Sciences and Technology of Kashmir, Srinagar, India; 2 Prince Fahd Bin Sultan Chair for Biomedical Research, Department of Medical Laboratory Technology, Faculty of Applied Medical Sciences, University of Tabuk, Tabuk, Saudi Arabia; 3 Division of Natural Resource Management, Faculty of Forestry, Sher-E-Kashmir University of Agricultural Sciences and Technology of Kashmir, Srinagar, India

**Keywords:** Aconitum heterophyllum, admet, androgen receptor, atisine, IL-1R1, molecular docking, molecular dynamics, network pharmacology

## Abstract

**Background:**

Lung and prostate cancers remain leading causes of cancer-related mortality worldwide, driven by inflammation-mediated tumour progression and hormone-dependent signalling. IL-1R1 promotes chronic inflammation, metastasis, and tumour survival, while androgen receptor (AR) signalling is a central driver of prostate cancer growth. Simultaneous targeting of inflammatory and androgenic pathways represents a promising strategy to overcome therapeutic resistance.

**Objectives:**

This study aimed to evaluate natural compounds derived from *Aconitum heterophyllum* (atisine, linoleic acid) and *Podophyllum hexandrum* (podophyllotoxin), as modulators of IL-1R1 and AR using integrated experimental and computational approaches.

**Methods:**

Phytochemicals were extracted from tuberous roots of *A. heterophyllum* and fruits of *P. hexandrum* using Soxhlet extraction and isolated by TLC and column chromatography. Cytotoxicity and long-term antiproliferative effects were assessed using MTT and colony formation assays in A549 (lung) and LNCaP (prostate) cancer cell lines. Computational analyses included ADMET profiling, molecular docking against IL-1R1 (PDB: 1ITB) and AR (PDB: 2YHD), 100 ns molecular dynamics simulations, MM-GBSA binding free-energy calculations, network pharmacology-based target and pathway enrichment analyses.

**Results:**

Podophyllotoxin exhibited potent cytotoxicity with IC_50_ values of 0.04 µM in both A549 and LNCaP cells, outperforming doxorubicin, while atisine showed moderate activity (IC_50_: 2.1–2.3 µM). Linoleic acid displayed weak anticancer activity (IC_50_ >100 µM). Colony formation assays confirmed strong suppression of clonogenic survival by podophyllotoxin and a moderate inhibitory effect by atisine. Docking studies suggested favorable binding affinities (−8.1 to −5.9 kcal/mol), and molecular dynamics simulations demonstrated stable protein–ligand complexes with low RMSD fluctuations. ADMET analysis supported favorable drug-likeness for atisine and podophyllotoxin. Network pharmacology identified 133 intersecting targets, with key hub genes including SRC, EGFR, and AKT1, enriched in PI3K-Akt and cancer-related signaling pathways.

**Conclusion:**

Atisine and podophyllotoxin exhibited significant anticancer activity and demonstrated favorable predicted interactions with the Interleukin-1 receptor type 1 (IL-1R1) and Androgen Receptor (AR), suggesting their potential as dual modulators that warrant further experimental validation. Their promising anticancer profile was supported by strong cytotoxicity, favorable predicted protein–ligand interactions, acceptable pharmacokinetic properties, and multitarget network pharmacology analyses. These findings provide a preliminary foundation for further optimization and *in vivo* validation toward the development of novel anticancer therapeutics targeting inflammation-driven and androgen-dependent cancers.

## 1 Background

Cancer continues to be a major global health concern, ranking second only to cardiovascular diseases in terms of mortality. In 2020, nearly 10 million people worldwide died from cancer, with lung and prostate cancers representing some of the most prevalent and therapeutically challenging forms due to drug resistance and metastatic spread (Apte and Voronov, 2008; Atanasov et al., 2021). These challenges highlight the urgent need for innovative therapeutic strategies that can intervene in key molecular pathways driving tumor progression.

Among these pathways, the roles of Interleukin-1 receptor type (IL-1R1) and androgen receptor (AR) signaling have gained considerable attention.Interleukin-1 receptor type 1 (IL-1R1) promotes tumor development by enhancing chronic inflammation, angiogenesis, and cell survival mechanisms (Choi et al., 2015; Cragg and Newman, 2013). Meanwhile, AR signaling remains central to prostate cancer progression, where its activation drives androgen-dependent growth and tumor maintenance (Dinarello., 2009; García et al., 2024). Simultaneous targeting of IL-1R1 and AR therefore offers an attractive strategy to counteract inflammation-driven and androgen-dependent cancers.

Natural products have long served as an invaluable source of anticancer agents due to their structural diversity, multitarget potential, and generally favorable safety profiles. Remarkably, nearly three-quarters of clinically approved anticancer drugs are derived from natural products or their structural analogs (Gordaliza et al., 2000; Lv et al., 2024). *Aconitum heterophyllum* Wall. ex Royle, known as “atis” in traditional medicine, is an endangered Himalayan herb used in Ayurveda and traditional Chinese medicine. It is known for its hepatoprotective, antimicrobial, antipyretic, and analgesic properties, and recent studies have suggested that atisine, one of its major diterpenoid alkaloids, possesses immunomodulatory and anticancer potential (Man et al., 2012; Mishra et al., 2022). Likewise, *Podophyllum hexandrum* Royle (Himalayan mayapple) is a rich source of the lignan Podophyllotoxin (PTOX), which serves as the scaffold for the clinically approved chemotherapeutics etoposide and teniposide. PTOX not only disrupts microtubule assembly but also demonstrates antiviral, immunosuppressive, and anti-inflammatory effects, and has been shown to interfere with AR signaling in prostate cancer (Nguyen et al., 2022; Nguyen et al., 2024; Nguyen et al., 2025).

Despite these promising activities, further development of natural metabolites requires a thorough understanding of their pharmacokinetic and safety properties. In this regard, computational strategies have become indispensable in modern drug discovery. Structure-based approaches such as molecular docking, molecular dynamics (MD) simulations, and ADMET (absorption, distribution, metabolism, excretion, and toxicity) analysis provide insights into drug-likeness, stability of ligand–protein complexes, and potential binding free energies (Nyirimigabo et al., 2015; Peña-Morán et al., 2016; Rai’dah et al., 2025). These techniques accelerate the identification of promising leads and reduce the reliance on costly and time-intensive experimental trials.

In the present study, we investigated two well-characterized phytoconstituents, atisine from *A. heterophyllum* and podophyllotoxin from *P. hexandrum*, as natural lead compounds targeting the Interleukin-1 receptor type 1 (IL-1R1) and Androgen Receptor (AR). In addition, linoleic acid was included because it was identified as one of the predominant constituents of the methanolic extract of *A. heterophyllum* by GC–MS analysis and was subsequently isolated through TLC-guided chromatographic fractionation. Rather than selecting linoleic acid based on its established anticancer activity, its inclusion was driven by its abundance in the bioactive extract and the need to determine whether a major extract-derived metabolite contributes to the observed biological effects. The anticancer potential of three compounds was assessed in A549 (lung cancer) and LNCaP (prostate cancer) cell lines using cytotoxicity and colony formation assays. Furthermore, ADMET profiling, molecular docking, and 100 ns molecular dynamics simulations were performed to evaluate their drug-likeness, binding affinity, and molecular interactions with IL-1R1 and AR, providing mechanistic insights into their potential as modulators of inflammatory and androgen signaling pathways in cancer.

## 2 Objectives

The present study was designed to investigate the anticancer potential of natural compounds derived from *A. heterophyllum* tuberous roots and *P. hexandrum* fruit, namely atisine and podophyllotoxin, along with linoleic acid, against lung and prostate cancers. Specifically, the objectives were to evaluate their cytotoxic and long-term antiproliferative effects in A549 and LNCaP cancer cell lines, assess their pharmacokinetic and drug-likeness properties through ADMET profiling, and elucidate their molecular interactions with key therapeutic targets interleukin-1 receptor type 1 (IL-1R1)and androgen receptor (AR) using molecular docking and molecular dynamics simulations. In addition, network pharmacology and pathway enrichment analyses were employed to uncover multitarget mechanisms, identify key hub genes, and define the signaling pathways involved in their anticancer activity, thereby providing mechanistic insights into their potential as dual modulators of inflammation-driven and androgen-dependent cancer progression.

## 3 Methods

### 3.1 Phytochemical extraction

Extraction was carried out using Soxhlet apparatus with solvents of increasing polarity: hexane, diethyl ether, ethyl acetate, methanol, and water, as described by (Rodríguez-López et al., 2021; Shen and Abate-Shen, 2010). The obtained extracts of tuberous roots of *A. heterophyllum* and fruits of *P. hexandrum* were filtered through Whatman No. 1 filter paper and concentrated to dryness using a rotary evaporator at 40 °C. The dried residues were weighed to determine the percentage yield and then stored at 5 °C for future analysis. All dried extracts were preserved in sterile, screw-capped amber bottles labelled appropriately and kept at 5 °C until further use.

### 3.2 TLC/column chromatography

To determine the optimal mobile phase for separating compounds, present in the required extracts, analytical Thin Layer Chromatography (TLC) was performed on silica gel pre-coated aluminium sheets (5 cm × 10 cm) (Macherey-Nagel & Co., Düren, Germany) using various solvent systems, as described by (Shukla et al., 2010). For *A. heterophyllum*, a mobile phase comprising methanol and chloroform in a 3:2 ratio was used. For *P. hexandrum* fruit extract, a mobile phase comprising hexane and methanol in a 3:1 ratio was standardised. After development in the respective mobile phases, the TLC plates were air-dried, and the bands were marked with a pencil. The retention factor (Rf) values were calculated for each spot using the formula: Rf = Distance travelled by the spot/Distance travelled by the solvent. Following analytical TLC, preparative TLC and column chromatography were employed to isolate larger quantities of the compounds for further bioactivity assessment and structural elucidation. In the case of *A. heterophyllum*, the visible bands were scraped from the preparative TLC plate and dissolved in methanol. The solution was centrifuged at 14,000 rpm for 10 min, and the resulting supernatant was transferred to a fresh microcentrifuge tube, dried, and stored for future use. For *P. hexandrum*, fractions were collected from a 50 mL column and concentrated using a rotary evaporator. TLC was again used to identify the compounds present in each fraction. The confirmed fractions were transferred to fresh tubes for further use.

### 3.3 Cell lines and culture conditions

Human lung carcinoma (A549) and prostate carcinoma (LNCaP) cell lines were procured from a certified cell repository. Cells were cultured in Dulbecco’s Modified Eagle’s Medium (DMEM, high glucose) supplemented with 10% fetal bovine serum (FBS) and 1% penicillin–streptomycin. Cultures were maintained at 37 °C in a humidified atmosphere of 5% CO_2_ and passaged regularly to ensure exponential growth.

### 3.4 Preparation of test compounds

Atisine, linoleic acid, and podophyllotoxin were isolated from the respective plant extracts by TLC-guided column chromatography. The identity and purity of the isolated compounds were confirmed by thin-layer chromatography (TLC) and structural characterization using ^1^H and ^13^C nuclear magnetic resonance (NMR) spectroscopy, and the obtained spectra were consistent with published literature. Doxorubicin hydrochloride (positive control) was obtained from a commercial supplier. Stock solutions of atisine, linoleic acid, and podophyllotoxin were prepared in dimethyl sulfoxide (DMSO), whereas doxorubicin was dissolved in sterile water. Working solutions were freshly prepared by diluting the stock solutions in complete culture medium, ensuring that the final concentration of DMSO did not exceed 0.5% (v/v) in any treatment.

### 3.5 MTT cytotoxicity assay

The cytotoxicity of the compounds was assessed using the 3-(4,5-dimethylthiazol-2-yl)-2,5-diphenyltetrazolium bromide (MTT) assay. Briefly, A549 and LNCaP cells were seeded in 96-well plates at a density of 5 × 10^3^ cells per well in 100 µL of complete medium and allowed to adhere overnight. Cells were then treated with varying concentrations of the test compounds for 24 h. Untreated cells served as negative control, while doxorubicin served as a positive control. After treatment, 20 µL of MTT solution (5 mg/mL in PBS) was added to each well and incubated for 4 h at 37 °C. The resulting formazan crystals were dissolved in 150 µL of DMSO, and absorbance was measured at 570 nm using a microplate reader, with a reference wavelength of 630 nm to correct background interference.

### 3.6 Data analysis

Percentage inhibition of cell viability was calculated relative to untreated control cells using the formula.

$$\text{Cell Viability }\left(\%\right)=\frac{\text{Absorbance of treated cells}}{\text{Absorbance of control cells}}\times 100$$

The IC_50_ values (concentration required to inhibit 50% cell viability) were determined using nonlinear regression analysis in GraphPad Prism 7. All experiments were performed in triplicate, and data were expressed as mean ± standard deviation (SD).

### 3.7 Colony forming unit assay

The Colony Forming Unit (CFU) assay was utilized to assess how the compounds influenced long-term cell viability and their ability to proliferate. LNCaP cells and A549 cells were placed in 12-well plates and allowed to grow until they reached 70%–75% confluency. They were then washed with PBS and treated with varying concentrations of the compounds (30, 40, and 50 μg/mL) alongside a DMSO vehicle control for a duration of 48 h. The concentrations used in the colony formation assay (30, 40, and 50 μg/mL) were selected based on preliminary cytotoxicity screening and were intended to represent sublethal doses that allowed sufficient cell survival for long-term clonogenic evaluation. Although the MTT assay concentrations are expressed in μM, the colony formation assay concentrations are reported in μg/mL because the compounds were prepared and administered as stock solutions based on mass concentration for prolonged treatment. The selected concentrations were chosen to maintain consistency with the preliminary optimization experiments while enabling accurate assessment of long-term antiproliferative activity. Post-treatment, viable cells from each well were counted using a hemocytometer, and equal numbers were reseeded into 10 cm culture dishes, where they were kept under standard growth conditions for 7–10 days to facilitate colony formation. Once colonies had formed, the culture media were carefully removed, and the cells were fixed with 100% methanol for 20 min, followed by a gentle rinse with distilled water. The plates were then stained with crystal violet (0.5%) for 5 min at room temperature, thoroughly washed to eliminate excess dye, and air-dried upside down on absorbent paper. Colonies larger than 0.5 mm were counted using ImageJ software (NIH, USA). The assay was performed in triplicate and independently repeated three times. Data were statistically analyzed and visualized using GraphPad Prism 7.0 (GraphPad Software, La Jolla, CA, USA).

### 3.8 ADMET prediction protocol

The ADMET (Absorption, Distribution, Metabolism, Excretion, and Toxicity) properties of the selected compounds were predicted using computational models implemented in the ADMETlab 2.0 web server (Singhuber et al., 2009). This tool provides a robust and comprehensive framework for evaluating drug-likeness and toxicity profiles. The molecular structures of the compounds, including atisine, linoleic acid, and podophyllotoxin, were first converted into SMILES format and then uploaded to the server for evaluation. Parameters such as molecular weight, volume, density, number of hydrogen bond acceptors (nHA), hydrogen bond donors (nHD), topological polar surface area (TPSA), number of rotatable bonds (nRot), logP (partition coefficient), logS (solubility), and potential for reactivity and toxicity were analysed.

### 3.9 Molecular docking

#### 3.9.1 Software tools

The molecular docking investigations were conducted using a wide range of software and resources, such as PyRx-Virtual Screening Tool, Discovery Studio Visualizer 2020, Autodock Vina, Pymol, MGL Tools, UCSF ChimeraX, Protein Data Bank (PDB), and PubChem. This approach ensured a thorough and diverse examination of the molecular interactions and binding strengths between the chosen compounds and the target proteins.

#### 3.9.2 Ligand preparation

The 3D structure of active compounds was obtained from the official website of the U.S. National Library of Medicine PubChem (https://pubchem.ncbi.nlm.nih.gov/). These structures were then imported into PyRx 0.8 using the open babel tool, followed by energy minimization (optimization). This optimization process considered fundamental parameters such as the element, hybridization, and connectivity, utilizing the Universal Force Field (Siegel et al., 2023). Subsequently, the ligand was converted to the AutoDock format (PDBQT).

#### 3.9.3 Target preparation

The three-dimensional (3D) crystal structures of the human androgen receptor (AR; PDB ID: 2YHD) (Sung et al., 2021) and the type-I interleukin-1 receptor (IL-1R1; PDB ID: 1ITB) (Vigers et al., 1997) were selected for molecular docking studies (Figure 1). The crystal structures were retrieved from the Research Collaboratory for Structural Bioinformatics (RCSB) Protein Data Bank (https://www.rcsb.org). Notably, PDB ID 1ITB corresponds to the type-I interleukin-1 receptor complexed with interleukin-1β (IL-1β). This receptor was selected because IL-1R1 is a key receptor involved in inflammation-associated signalling pathways implicated in cancer progression, making it a biologically relevant therapeutic target identified through network pharmacology analysis. Prior to docking, the receptor structures were prepared using Discovery Studio Visualizer 2020. The co-crystallized cytokine (IL-1β) from PDB ID 1ITB and the co-crystallized ligand from PDB ID 2YHD were removed prior to receptor preparation. Water molecules and non-essential heteroatoms were deleted, while polar hydrogen atoms and Kollman charges were added using AutoDock Tools 1.5.6 before conversion to PDBQT format. To validate the docking protocol, the co-crystallized ligand was re-docked into the receptor binding pocket, and the resulting pose showed good agreement with the experimental crystal structure (RMSD <2.0 Å), confirming the reliability of the docking procedure. The prepared receptor structures were subsequently energy-minimized and used for molecular docking studies. Molecular docking simulations of the selected compounds were performed using AutoDock Vina 1.1.2 implemented in PyRx 0.8. (Wang et al., 2018).

**FIGURE 1:**
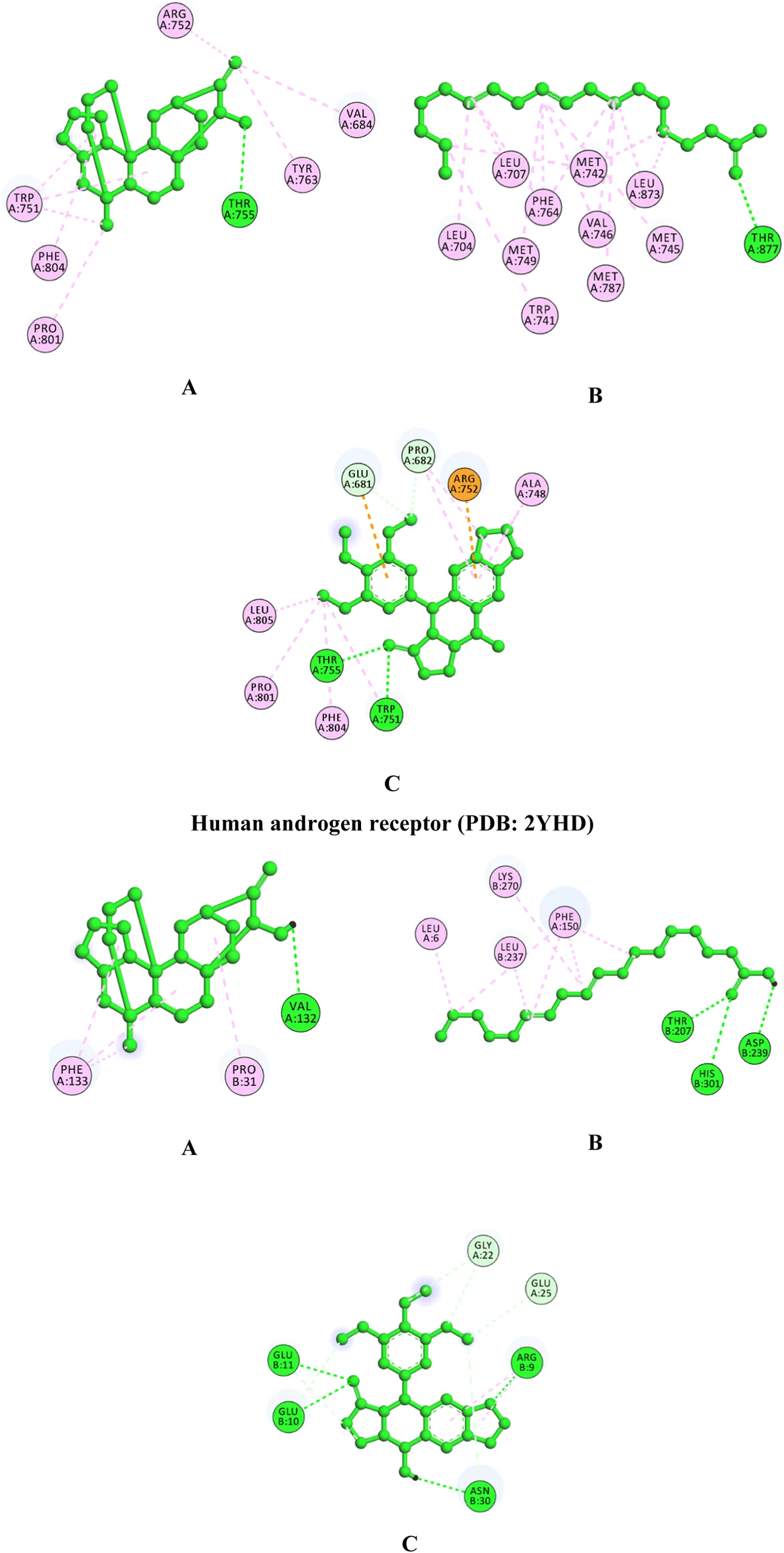
2D docking poses of active compounds **(A)** Atisine **(B)** Linoleic acid **(C)** Podophyllotoxin with AR and IL-1R1 respectively.

#### 3.9.4 Docking procedure

The purified structures of the targets were loaded to docking software PyRx 0.8 using a load molecule option from the File tool bar. The receptor structure then converted to AutoDock macromolecule (PDBQT format) using the right-click option. Binding affinity studies were performed using Vina Wizard Tool in PyRx 0.8. Molecules (PDBQT Files), all three compounds (atisine, podophyllotoxin and linoleic acid) as well as the targets were selected for docking. For molecular docking simulation, the three-dimensional grid box (size_x = 54.30 Å, size_y = 42.50 Å, size_z = 54.81 Å) for 2YHD and (size_x = 101.82 Å, size_y = 60.03 Å, size_z = 44.95 Å) for 1ITB was designed using AutoDock tool 1.5.6 with exhaustiveness value of 8. After selecting molecules, the active amino acid residues were selected to define the cavity with the help of the toggle selection spheres option given in PyRx. To fully occupy all the active binding sites and crucial residues, the grid box was appropriately positioned. Following this alignment, the ligands and targets underwent docking procedures to determine their mutual affinity. The docking grid was centered on the experimentally characterized ligand-binding region identified from the crystal structures and corresponding binding cavity analysis to ensure accurate sampling of the active site.

### 3.10 Identification of cavity and active amino acid residues

The identification of the binding cavity and key active amino acid residues was performed using Discovery Studio Visualizer (version 19.1.0.18287). The three-dimensional structure of the target protein was imported, and the “Define and Edit Binding Site” tool was used to predict potential cavities based on surface topology and volume. Among the identified pockets, the most probable active site was selected based on size, depth, and accessibility. Additionally, the co-crystallized ligand was used as a guide to refine the binding site location. The interacting amino acid residues within the selected cavity were highlighted, and key residues involved in hydrogen bonding, hydrophobic contacts, and electrostatic interactions were documented. These identified residues were further used to define the grid box center for molecular docking studies to ensure accurate ligand placement within the active site.

### 3.11 Molecular dynamics (MD) simulations

Molecular dynamics (MD) simulations were conducted using Desmond, developed by Schrödinger LLC, to assess the dynamic behavior and binding stability of the androgen receptor (AR) complexed with Podophyllotoxin and the interleukin-1 receptor type 1 (IL-1R1) complexed with atisine over a 100-nanosecond (ns) period. The receptor-ligand complexes, derived from prior docking studies, were subjected to MD simulations to evaluate binding under physiological conditions, employing Newton’s classical equations of motion. Protein-ligand systems were prepared using the Protein Preparation Wizard in Maestro to optimize structures, addressing steric clashes, undesirable interactions, and structural distortions. The System Builder tool constructed simulation systems with the TIP3P water model in an orthorhombic box, utilizing the OPLS_2005 force field. The systems were neutralized with counter ions and supplemented with 0.15 M sodium chloride to mimic physiological conditions. Simulations were run at 300 K and 1 atm, with trajectories recorded every 100 picoseconds (ps). Stability of the protein-ligand complexes was analyzed *via* Root Mean Square Deviation (RMSD), confirming consistent binding interactions throughout the simulation period (Wen et al., 2019; Xie et al., 2025).

### 3.12 Prime MM-GBSA analysis

The binding free energy (ΔG_bind) of the docked complexes (Podophyllotoxin-AR and Atisine-IL-1R1) was calculated using the Molecular Mechanics Generalized Born Surface Area (MM-GBSA) method within the Prime module of Schrödinger. The OPLS_2005 force field and VSGB implicit solvent model were applied, with rotamer exploration to ensure accurate conformational sampling. Frames from the MD trajectories were analyzed at 10 ns intervals. The binding free energy was determined using the equation:

$$\Delta G_{\text{bind}}=G_{\text{complex}}\;–\;\left(G_{\text{protein}}+G_{\text{ligand}}\right)$$

Where, ΔG_bind = binding free energy, G_complex = free energy of the complex, G_protein = free energy of the target protein, and G_ligand = free energy of the ligand (You et al., 1984).

### 3.13 Network pharmacology

#### 3.13.1 Prediction of disease targets of active compounds

The 3D structures of the active constituent’s atisine, linoleic acid, and podophyllotoxin were drawn in ChemBioDraw and saved as “sdf” file format. This file was then imported into Swiss target prediction database (https://www.swisstargetprediction.ch) (Zi et al., 2019), inputting the protein names with the species limited to “*Homo sapiens*,” and we could receive their official symbol. After these operations, protein information of compound targets and known targets was obtained.

#### 3.13.2 Gene screening of disease related targets

Using “Lung and prostate cancer” as the search keywords, we searched OMIM (https://www.omim.org/, accessed on 20 August 2024), Gene Cards database (https://www.genecards.org/, accessed on 20 August 2024) [29] to excavate potential targets associated with cancer. Venny 2.1.0 (https://bioinfogp.cnb.csic.es/tools/venny/) software was used to identify the targets of active compounds against cancer (Stelzer et al., 2016).

#### 3.13.3 GO, KEGG and functional enrichment analyses

Functional enrichment analysis of the overlapping target genes was performed using ShinyGO v0.82 to investigate their potential biological significance. Gene Ontology (GO) enrichment analysis was conducted across the three principal GO categories: Biological Process (BP), Molecular Function (MF), and Cellular Component (CC). In addition, Kyoto Encyclopedia of Genes and Genomes (KEGG) pathway enrichment analysis was performed to identify significantly enriched signaling pathways associated with the intersecting targets. Enrichment results were ranked according to the number of genes involved in each term or pathway and only significantly enriched terms (P < 0.05) were considered for further interpretation. Histograms and bar plots illustrating the GO and KEGG enrichment analyses were generated using ShinyGO. The top ten significantly enriched GO terms and KEGG pathways, along with the corresponding core target genes, were selected for visualization and subsequent analysis.

#### 3.13.4 Network construction

The next phase is figuring out the cellular mechanism, which is accomplished by creating a compound-target network with Cytoscape software. The compound-target network helps in understanding and analyzing the process of the component-target interaction, as well as the pathway associated. In this network, nodes represent the active ingredients and targets, while edges depict their interactions. The network analyzer function was used to determine the network’s basic properties and to calculate the degree that sheds light on the connectedness of the network and the importance of the connections between the genes, active components, and their associated pathways.

#### 3.13.5 Hub gene identification and PPI network

To further identify the core regulatory targets, PPI analysis was performed by submitting overlapping targets of active compounds in cancer to the STRING 11.5 database (https://string-db.org/, accessed on 20 August 2024). The species type was set to “*H. sapiens*,” the minimum interaction threshold was set to “High confidence” >0.7, and the rest was set as the default. Finally, the PPI result was imported into Cytoscape 3.10.1 software to construct and analyse the PPI network. The nodes of the network were analyzed for their topological features and a range of possible targets were selected by taking into account three parameters, namely “degree,” “betweeness centrality,” and “closeness centrality.” These three parameters were used to estimate the topological properties of the nodes. The CytoHubba plugin was employed to detect hub genes and nodes with significant levels within the network. The presence of the highest degree emphasizes the substantial correlations between the genes under consideration.

## 4 Results

### 4.1 Validation of structural elucidation

The isolated compounds were confirmed using TLC (Supplementary Figure S1) 1H and 13C NMR spectra, matching known chemical signatures from literature (Supplementary Figures S2–S4).

### 4.2 MTT assay of compounds

The comparative cytotoxic potential of atisine, podophyllotoxin, linoleic acid, and doxorubicin was evaluated against A549 (lung cancer) and LNCaP (prostate cancer) cell lines (Table 1). The results revealed distinct inhibitory profiles, highlighting the differential efficacy of the tested compounds. Atisine demonstrated a clear dose-dependent cytotoxic effect in both cancer cell lines. At a concentration as low as 0.5 µM, inhibition was already evident (14.8% in A549; 12.5% in LNCaP), progressively increasing to ∼85% at 10 µM. The calculated IC_50_ values (2.1 µM for A549 and 2.3 µM for LNCaP) suggest moderate potency. Podophyllotoxin emerged as the most potent compound, exhibiting strong inhibition even at nanomolar levels. At 0.01 µM, it showed ∼17% inhibition, rapidly escalating to over 90% inhibition at 0.5 µM. The extremely low IC_50_ value (0.04 µM for both A549 and LNCaP) indicates exceptional cytotoxic efficacy. Linoleic acid exhibited weak anticancer activity, with maximum inhibition of 37.2% in A549 cells and 34.9% in LNCaP cells at 100 µM. Therefore, the IC_50_ value could not be accurately determined within the tested concentration range and was considered to be > 100 µM. Doxorubicin (positive control) displayed high potency with IC_50_ values of 0.25 µM (A549) and 0.22 µM (LNCaP). The inhibition profile was consistent and comparable to Podophyllotoxin.

**TABLE 1:** Comparative cytotoxic potential of the isolated compounds against A549 (lung cancer) and LNCaP (prostate cancer) cell lines as determined by MTT assay.

Compound	Conc. (µM)	% Inhibition		IC_50_ (µM)	
		(A549)	(LNCaP)	(A549)	(LNCaP)
Atisine	0.5	14.8 ± 2.0	12.5 ± 2.1	2.1	2.3
	1	35.7 ± 1.8	33.8 ± 1.9		
	2.5	57.3 ± 1.5	55.1 ± 1.7		
	5	76.2 ± 1.2	73.6 ± 1.3		
	10	87.6 ± 1.1	85.4 ± 1.2		
Podophyllotoxin	0.01	17.5 ± 2.3	15.3 ± 2.4	0.04	0.04
	0.05	60.2 ± 1.9	57.7 ± 2.0		
	0.1	78.3 ± 1.4	76.0 ± 1.5		
	0.5	90.8 ± 1.0	88.7 ± 1.1		
	1	95.4 ± 0.8	93.8 ± 0.9		
Linoleic acid	5	7.7 ± 2.4	6.2 ± 2.5	>100	>100
	10	12.4 ± 2.1	10.8 ± 2.2		
	25	21.5 ± 1.9	19.3 ± 2.0		
	50	30.8 ± 1.7	28.5 ± 1.8		
	100	37.2 ± 1.6	34.9 ± 1.7		
Doxorubicin (control)	0.05	22.4 ± 2.0	20.8 ± 2.1	0.25	0.22
	0.1	48.6 ± 1.7	45.9 ± 1.8		
	0.25	71.3 ± 1.5	68.2 ± 1.6		
	0.5	86.5 ± 1.2	84.1 ± 1.3		
	1	94.2 ± 0.9	92.7 ± 1.0		

### 4.3 Colony forming unit assay

The long-term antiproliferative effects of linoleic acid, atisine, and podophyllotoxin were evaluated using a colony formation assay in A549 and LNCaP cells. Representative colony images are shown in Figures 2, 3, while quantitative analyses are presented in Figures 4, 5. In A549 cells, linoleic acid produced only a slight concentration-dependent reduction in colony formation, indicating a limited inhibitory effect on clonogenic survival. In contrast, atisine caused a moderate decrease in both colony number and colony size, demonstrating partial suppression of long-term proliferative capacity. Podophyllotoxin exhibited the most pronounced inhibitory effect, producing a marked concentration-dependent reduction in colony formation with only a few small colonies remaining at the highest concentration tested (Figure 2). Quantitative analysis confirmed these observations (Figure 4). Atisine significantly reduced colony formation compared with the untreated control (p < 0.01), whereas podophyllotoxin produced a highly significant inhibition of clonogenic survival (*p < 0.001). Linoleic acid showed only a slight reduction in colony formation, which was not statistically significant (ns), indicating relatively weak long-term antiproliferative activity.

**FIGURE 2:**
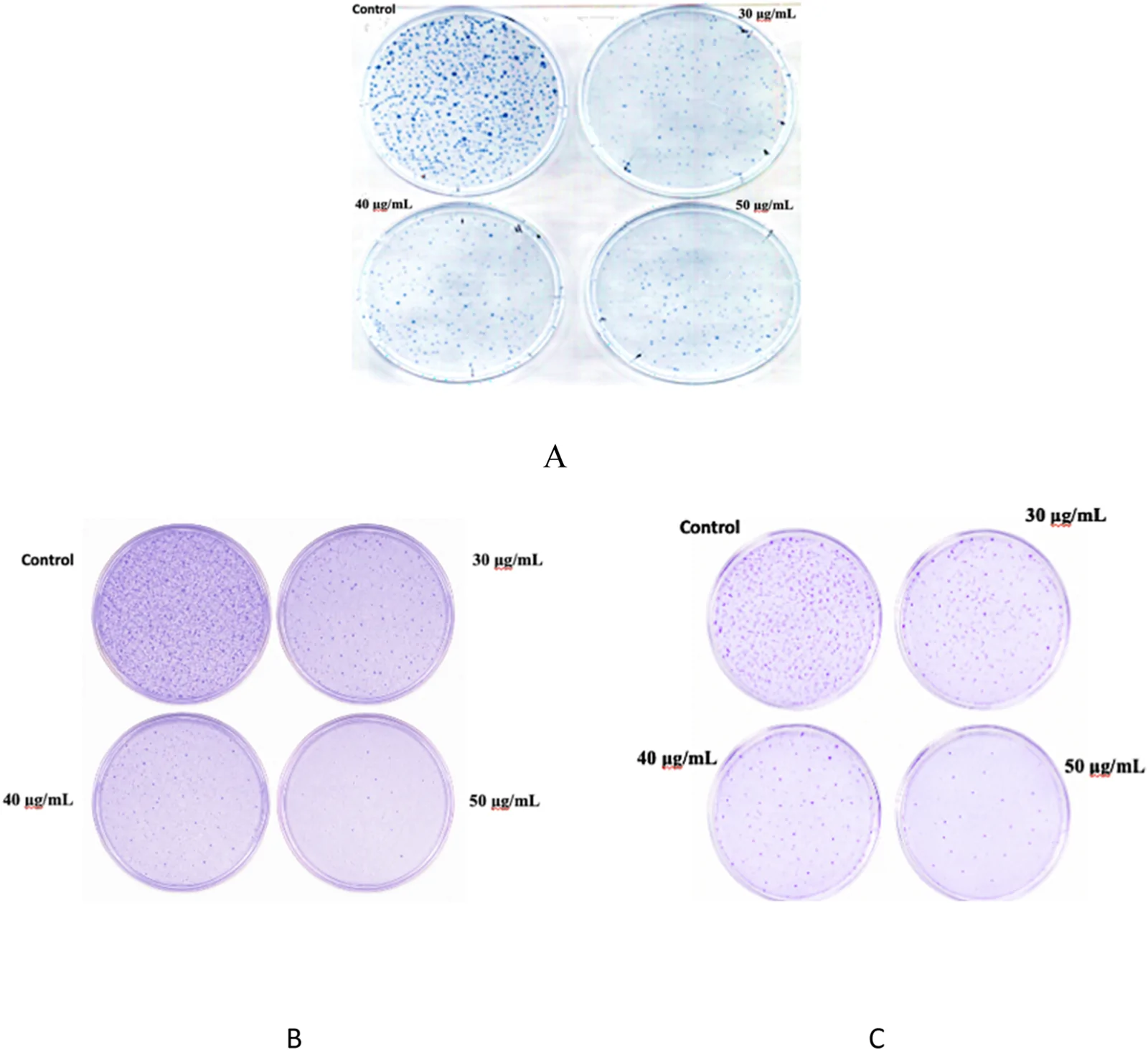
Colony formation assay showing the long-term effects of the tested compounds on clonogenic survival in A549 cells **(A)** Linoleic acid treatment shows a mild reduction in colony number compared with control, indicating a limited effect on clonogenic growth. **(B)** Atisine treatment results in a moderate decrease in colony number and size, reflecting partial inhibition of long-term proliferative capacity **(C)** Podophyllotoxin treatment causes a pronounced suppression of colony formation, with sparse and small colonies, demonstrating strong inhibition of clonogenic survival.

**FIGURE 3:**
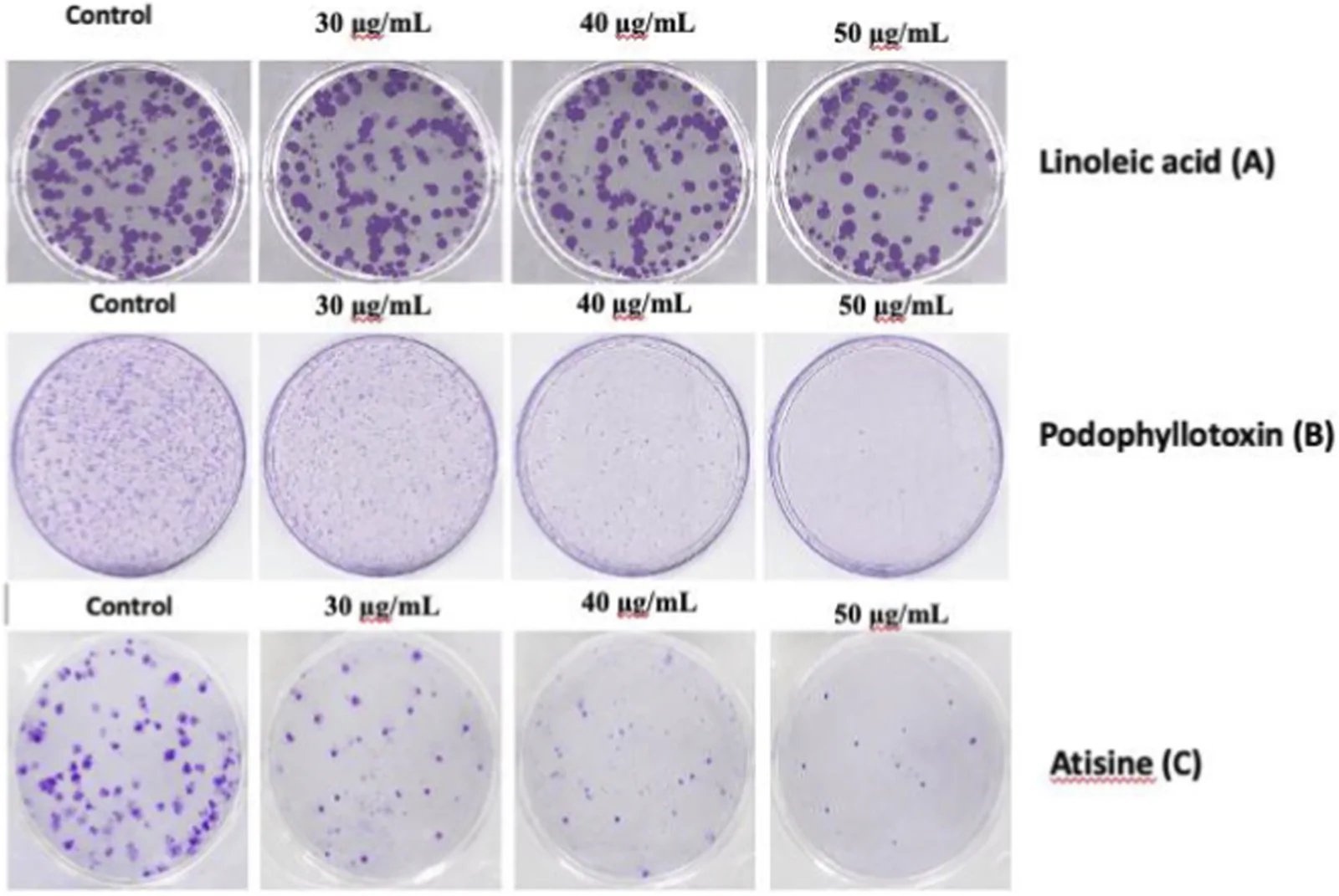
Colony formation assay showing the long-term effects of the tested compounds on clonogenic survival in LNCaP cells. **(A)** Linoleic acid treatment resulted in only a mild reduction in colony number compared with the untreated control, indicating a limited inhibitory effect on the clonogenic growth of LNCaP cells. **(B)** Podophyllotoxin treatment produced a marked concentration-dependent inhibition of colony formation, with sparse and smaller colonies observed at higher concentrations, indicating potent suppression of clonogenic survival in LNCaP cells **(C)** Atisine treatment caused a moderate concentration-dependent decrease in both colony number and colony size, demonstrating partial suppression of long-term proliferative capacity.

**FIGURE 4:**
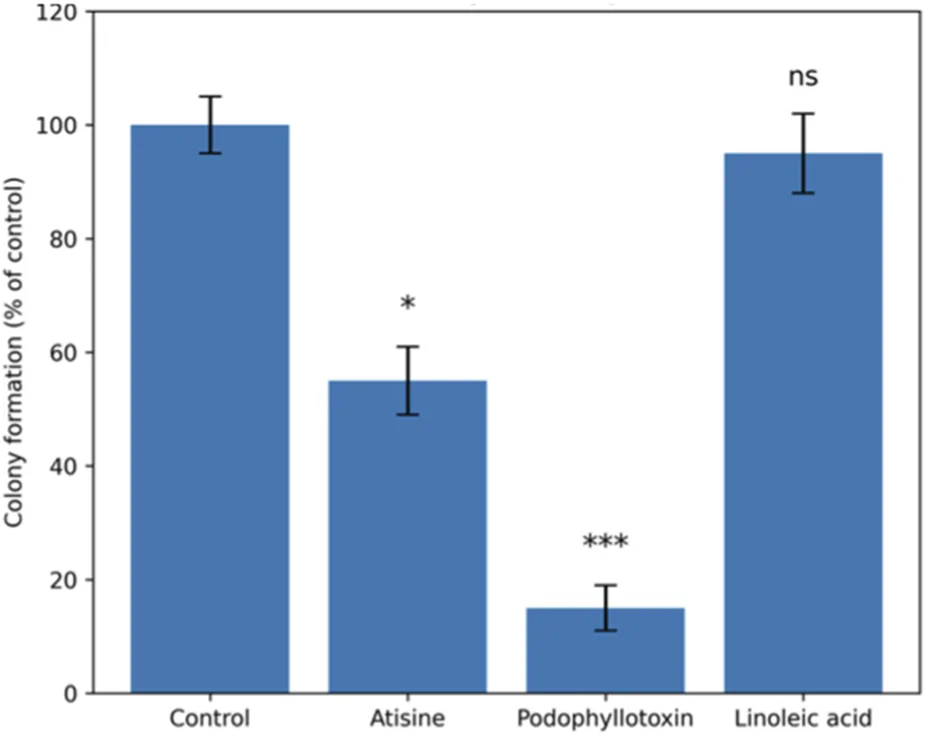
Quantitative analysis of colony formation following treatment with atisine, podophyllotoxin, and linoleic acid. Data are expressed as percentage of colony formation relative to control (mean ± SD). Atisine significantly reduced clonogenic survival (*p < 0.05), podophyllotoxin caused a highly significant inhibition (***p < 0.001), while linoleic acid showed no significant effect (ns) compared with control.

**FIGURE 5:**
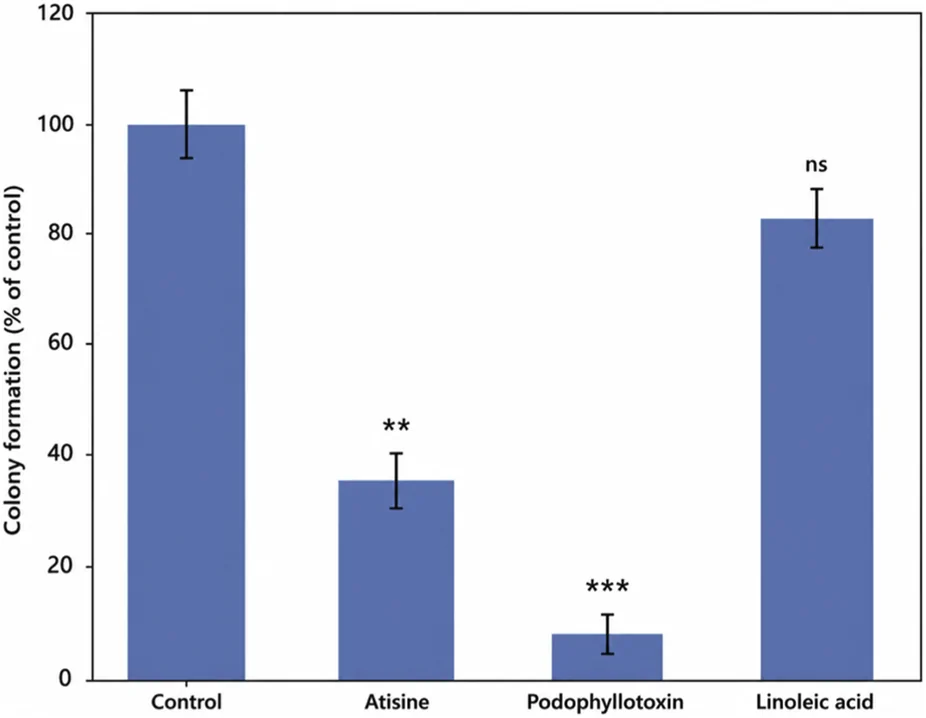
Quantitative analysis of colony formation in LNCaP cells following treatment with the investigated compounds. Colony formation was quantified using ImageJ software and expressed as the percentage of colony formation relative to the untreated control group (mean ± SD, *n* = 3). Atisine significantly reduced clonogenic survival (**p < 0.01), while podophyllotoxin exhibited the strongest inhibitory effect (***p < 0.001). In contrast, linoleic acid produced only a slight reduction in colony formation that was not statistically significant (ns) compared with the control group. Statistical significance was determined using one-way ANOVA followed by Tukey’s multiple comparison test.

A similar trend was observed in LNCaP cells (Figure 3). Linoleic acid resulted in only a mild reduction in colony number across the tested concentrations, whereas atisine induced a concentration-dependent decrease in both colony number and colony size, reflecting moderate inhibition of clonogenic growth. Podophyllotoxin again demonstrated the greatest inhibitory activity, causing a marked concentration-dependent suppression of colony formation and leaving only sparse colonies at the highest concentration. The quantitative analysis of LNCaP cells (Figure 5) corroborated the qualitative observations. Atisine significantly suppressed colony formation (p < 0.01), while podophyllotoxin exhibited the strongest inhibitory effect on clonogenic survival (*p < 0.001). In contrast, linoleic acid produced only a modest reduction in colony formation that was not statistically significant compared with the untreated control (ns). Overall, these findings demonstrate that podophyllotoxin possesses the most potent long-term antiproliferative activity, followed by atisine, whereas linoleic acid exhibits comparatively weak effects on clonogenic survival in both A549 and LNCaP cells.

### 4.4 ADMET and drug-likeness evaluation

The ADMET analysis of Podophyllotoxin, Atisine, and Linoleic acid revealed distinct pharmacokinetic and drug-likeness profiles (Tables 2–5). Podophyllotoxin exhibited favorable drug-likeness (QED = 0.763) with compliance to Lipinski, Pfizer, and GSK rules, a moderate TPSA (92.68 Å^2^), and good solubility (logS = −3.90), although its high plasma protein binding (97.41%) may restrict free drug concentration. Atisine also showed good drug-likeness (QED = 0.683) and low TPSA (32.7 Å^2^), indicating better membrane permeability; however, it was predicted as a P-glycoprotein substrate, suggesting possible efflux and reduced bioavailability despite moderate plasma protein binding (66.4%). Linoleic acid, in contrast, showed poor drug-likeness (QED = 0.318) with violations of Pfizer and GSK rules, low solubility (logS = −5.42), and excessive lipophilicity (logP = 6.95), consistent with its classification as a fatty acid rather than a conventional drug scaffold. Distribution studies further indicated that podophyllotoxin and atisine may penetrate the blood–brain barrier, whereas Linoleic acid lacked CNS penetration. Collectively, these findings suggest that podophyllotoxin and atisine display more favorable ADMET properties for therapeutic development, while Linoleic acid, due to its physicochemical limitations, may serve better as a supportive bioactive compound rather than a lead drug candidate.

**TABLE 2:** Physicochemical properties of compounds.

Compound	Molecular weight	TPSA	HBA	HBD	RotB	logP	logS
Podophyllotoxin	414.13	92.68	8	1	4	1.729	−3.904
Atisine	343.25	32.7	3	1	0	1.809	−2.439
Linoleic acid	280.24	37.3	2	1	14	6.953	−5.426

**TABLE 3:** Drug-likeness properties of compounds.

Compound	QED	NPscore	Lipinski rule	Pfizer rule	GSK rule	Golden triangle	Chelator rule
Podophyllotoxin	0.763	1.515	Accepted	Accepted	Accepted	Accepted	0
Atisine	0.683	2.963	Accepted	Accepted	Accepted	Accepted	0
Linoleic acid	0.318	1.187	Accepted	Violated	Violated	Violated	0

**TABLE 4:** Absorption and bioavailability of compounds.

Compound	Caco-2	MDCK	PAMPA	P-gp substrate	P-gp Inhibitor	HIA	F20%	F30%/F50%
Podophyllotoxin	−5.073	−4.762	0.301	No	No	Low	0.012	0.0/0.098
Atisine	−5.074	−4.881	0.989	Yes	No	Low	0.042	0.318/0.864
Linoleic acid	−5.051	−4.843	0.025	No	No	Low	0.148	0.388/0.608

**TABLE 5:** Distribution and metabolism of compounds.

Compound	PPB (%)	VDss	BBB	Fu (%)	OATP1B1 inhibitor	OATP1B3 inhibitor
Podophyllotoxin	97.41	−0.295	Yes	2.82	0.258	0.565
Atisine	66.421	0.049	Yes	34.031	0.988	0.499
Linoleic acid	97.489	−0.403	No	1.574	0.005	0.556

### 4.5 Molecular docking and binding affinity analysis

Molecular docking analyses were conducted to evaluate the binding affinities and interaction profiles of three compounds—atisine, linoleic acid, and podophyllotoxin—against the Human androgen receptor (AR; PDB: 2YHD) and type-I Interleukin-1 receptor (IL-1R1; PDB:1ITB), as shown in Table 6 and illustrated in Figures 1, 6–8. Among the three investigated compounds, Podophyllotoxin and Atisine exhibited the strongest binding affinities toward AR and IL-1R1, respectively, whereas Linoleic acid demonstrated comparatively weaker binding, consistent with its lower *in vitro* cytotoxic activity. For AR, binding affinities ranged from −8.0 to −6.1 kcal/mol, with podophyllotoxin exhibiting the highest affinity (−8.0 kcal/mol), followed by atisine (−7.2 kcal/mol) and linoleic acid (−6.1 kcal/mol). Podophyllotoxin’s interaction with AR included hydrophobic contacts with GLU681, PRO682, ARG752, and PHE804, and hydrogen bonds with TRP751, ARG752, and THR755. Atisine formed hydrophobic interactions with PHE804 and LEU805, and hydrogen bonds with THR755 and ASN756. Linoleic acid showed weaker interactions, primarily hydrophobic with LEU704, TRP741, and THR877, and a hydrogen bond with THR877. For Interleukin-1 receptor, binding affinities ranged from −8.1 to −5.9 kcal/mol, with atisine achieving the highest score (−8.1 kcal/mol), followed by podophyllotoxin (−7.6 kcal/mol) and linoleic acid (−5.9 kcal/mol). Atisine’s interaction with IL-1R1 involved hydrophobic contacts with GLU25 and PRO131, and a hydrogen bond with VAL132. Podophyllotoxin formed hydrophobic interactions with ARG9 and multiple hydrogen bonds with ARG9, GLU10, GLU11, and ASN30. Linoleic acid interacted hydrophobically with LEU6, PHE150, and LEU237 and formed hydrogen bonds with THR207, GLN236, and ASN299.

**TABLE 6:** Molecular docking study of the active compounds against the targets.

Targets	Compounds	Binding affinity	Hydrophobic Interactions	Hydrogen bonding
AR	Atisine	−7.2	PHE804, LEU805	THR755, ASN756
	Linoleic_acid	−6.1	LEU704, LEU707, TRP741, MET745, VAL746, PHE764, LEU873	THR877
	Podophyllotoxin	−8	GLU681, PRO682, ARG752, PHE804	TRP751, ARG752, THR755
IL-1R1	Atisine	−8.1	GLU25, PRO131	VAL132
	Linoleic_acid	−5.9	LEU6, PHE150, LEU237	THR207, GLN236, ASN299
	Podophyllotoxin	−7.6	ARG9	ARG9, GLU10, GLU11, ASN30

**FIGURE 6:**
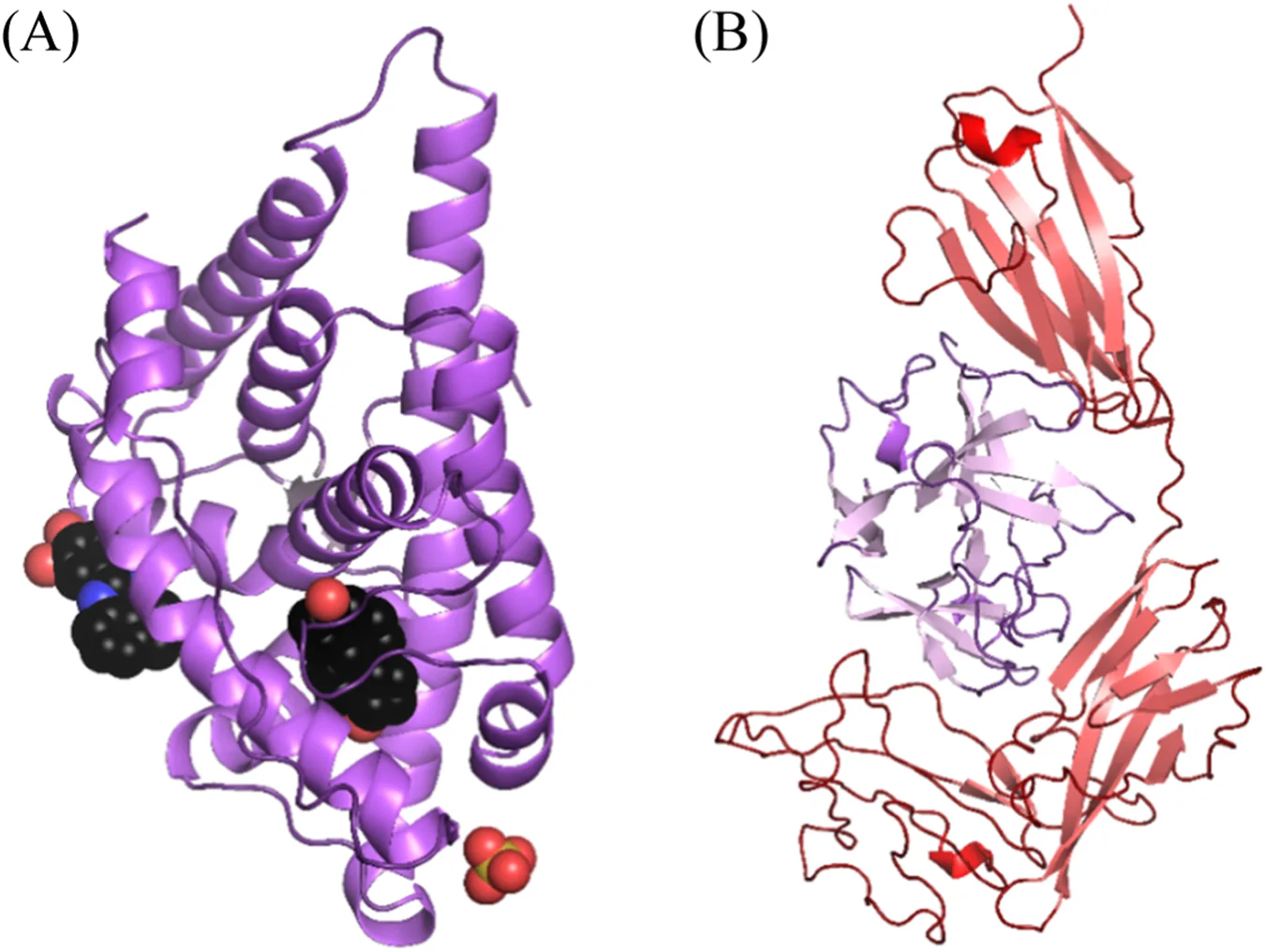
Protein Targets **(A)** Human androgen receptor (PDB: 2YHD) **(B)** Type-1 Interleukin-1 receptor (PDB: 1ITB).

**FIGURE 7:**
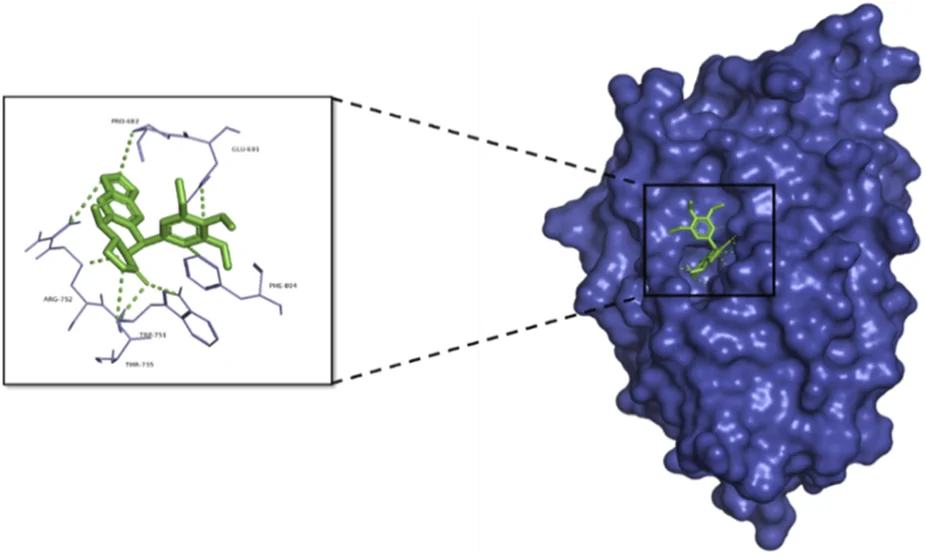
Amino acid interactions formed between docked complexes of Podophyllotoxin with Human androgen receptor (PDB: 2YHD).

**FIGURE 8:**
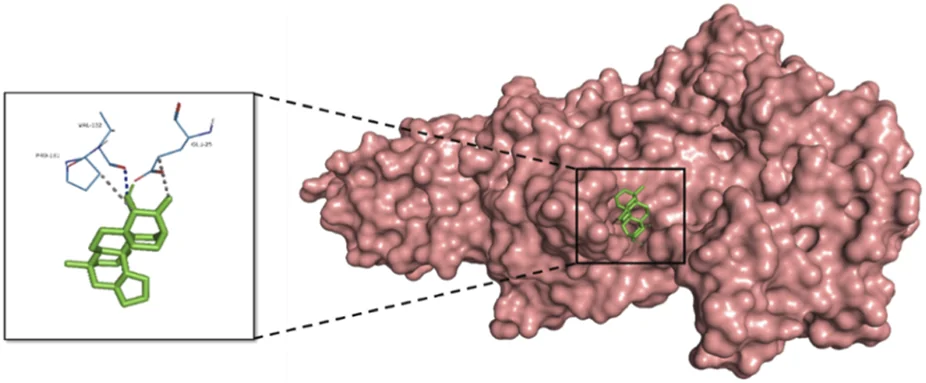
Amino acid interactions formed between docked complexes of Atisine with Type-1 Interleukin-1 receptor (PDB: 1ITB).

### 4.6 Molecular dynamics (MD) simulations

Based on the molecular docking results, podophyllotoxin and atisine were selected for molecular dynamics (MD) simulations to evaluate further their stability and binding interactions with the respective protein targets. A 100 ns simulation was performed for each complex, and the stability, structural fluctuations, secondary structure composition, and protein–ligand interactions were analysed using the trajectory data (Figure 9).

**FIGURE 9:**
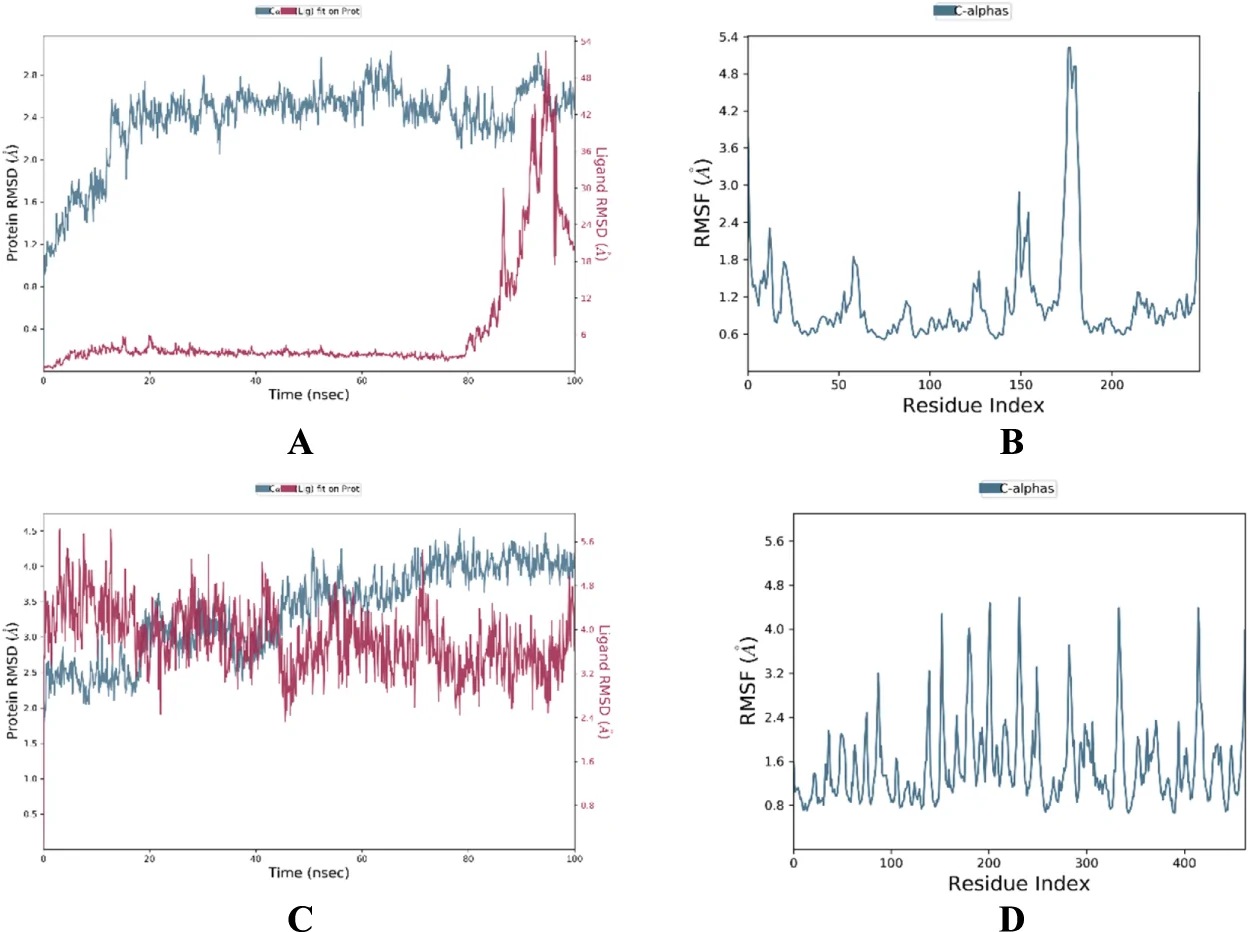
RMSD/RMSF values during molecular dynamics simulation **(A,B)** AR-Podophyllotoxin complex, (**C,D**) IL-1R1–Atisine complex.

For the Podophyllotoxin-AR complex, the RMSD of the Cα atoms of the protein remained stable throughout the 100 ns simulation, fluctuating within the range typically acceptable for small globular proteins (Figure 9A). The ligand RMSD also indicated a stable binding mode, with minor deviations during the initial phase, which later stabilized, confirming the ligand’s consistent accommodation within the binding pocket. The RMSF analysis (Figure 9B) showed that terminal and loop residues exhibited higher flexibility, while the core secondary structure elements remained rigid. For the Atisine- IL-1R1 complex, the protein backbone RMSD values were stable after equilibration, indicating a converged trajectory (Figure 9C). The ligand RMSD remained consistent, reflecting stable accommodation within the active site throughout the 100 ns simulation. The RMSF profile (Figure 9D) showed moderate flexibility at specific loop regions, whereas structured domains displayed minimal fluctuations, consistent with protein rigidity. The secondary structure composition of Podophyllotoxin revealed that α-helices contributed 52.30%, β-strands 1.72%, and other structural elements 46% of the total, highlighting the dominance of helical conformations during the simulation (Supplementary Figure S5). Interestingly, the secondary structure analysis of Atisine revealed a higher proportion of β-strands (41.79%) with minimal α-helical content (0.24%), demonstrating that the protein maintained a β-strand–rich conformation during the simulation (Supplementary Figure S6). Protein–ligand interaction profiling (Supplementary Figure S7A) showed persistent hydrogen bonding, hydrophobic contacts, and water bridge interactions involving residues critical for stable binding, suggesting that Podophyllotoxin maintains robust intermolecular interactions over the simulation trajectory.

For Atisine- IL-1R1 complex the protein–ligand interaction mapping (Supplementary Figure S7B) highlighted stable hydrogen bonds, water-mediated bridges, and hydrophobic interactions involving residues that contributed to ligand stabilization. Additionally, the full description of ligand properties is shown in (Supplementary Figures S8A,B). Overall, both complexes demonstrated stable RMSD and RMSF behavior, maintained secondary structure integrity, and exhibited persistent protein–ligand interactions during the 100 ns simulations. These results strongly support the favorable binding stability and structural compatibility of Podophyllotoxin and Atisine with their respective targets, offering molecular-level insights into their therapeutic potential. Additionally, the minimized binding free energies of the complexes were determined using the MM-GBSA method, as detailed in (Table 7).

**TABLE 7:** Binding-free energies (kcal/mol) obtained by MM–GBSA of podophyllotoxin and atisine

Compound	Time (ns)	MMGBSA-dG-binding energy	MMGBSA-dG-bind in Coulomb	MMGBSA-dG-bind (NS)	MMGBSA-dG bind (NS)-Coulomb
Podophyllotoxin	0	−63.627053	−7.920549	−66.923806	−8.077138
Podophyllotoxin	100	−30.958436	1.733699	−31.826946	1.515065
Atisine	0	−71.246033	−7.232856	−73.188014	−7.128057
Atisine	100	−49.766928	−2.613812	−51.762202	−2.529501

### 4.7 Network pharmacology

#### 4.7.1 Active ingredients screening and collection of disease-relevant targets

In the context of this study, the network pharmacology approach was employed to uncover the mechanism of action and potential targets associated with active constituents in its application for cancer treatment. As illustrated in Figure 10, by searching the public databases (Swiss target prediction, OMIM, Gene Cards), confining the result to “*H. sapiens*,” 327 compound‐related targets and 1,299 cancer‐related targets were collected, respectively. The utilization of Venn 2.0.2 facilitated the calculation of the intersection between compound targets and disease targets, resulting in 1

**FIGURE 10:**
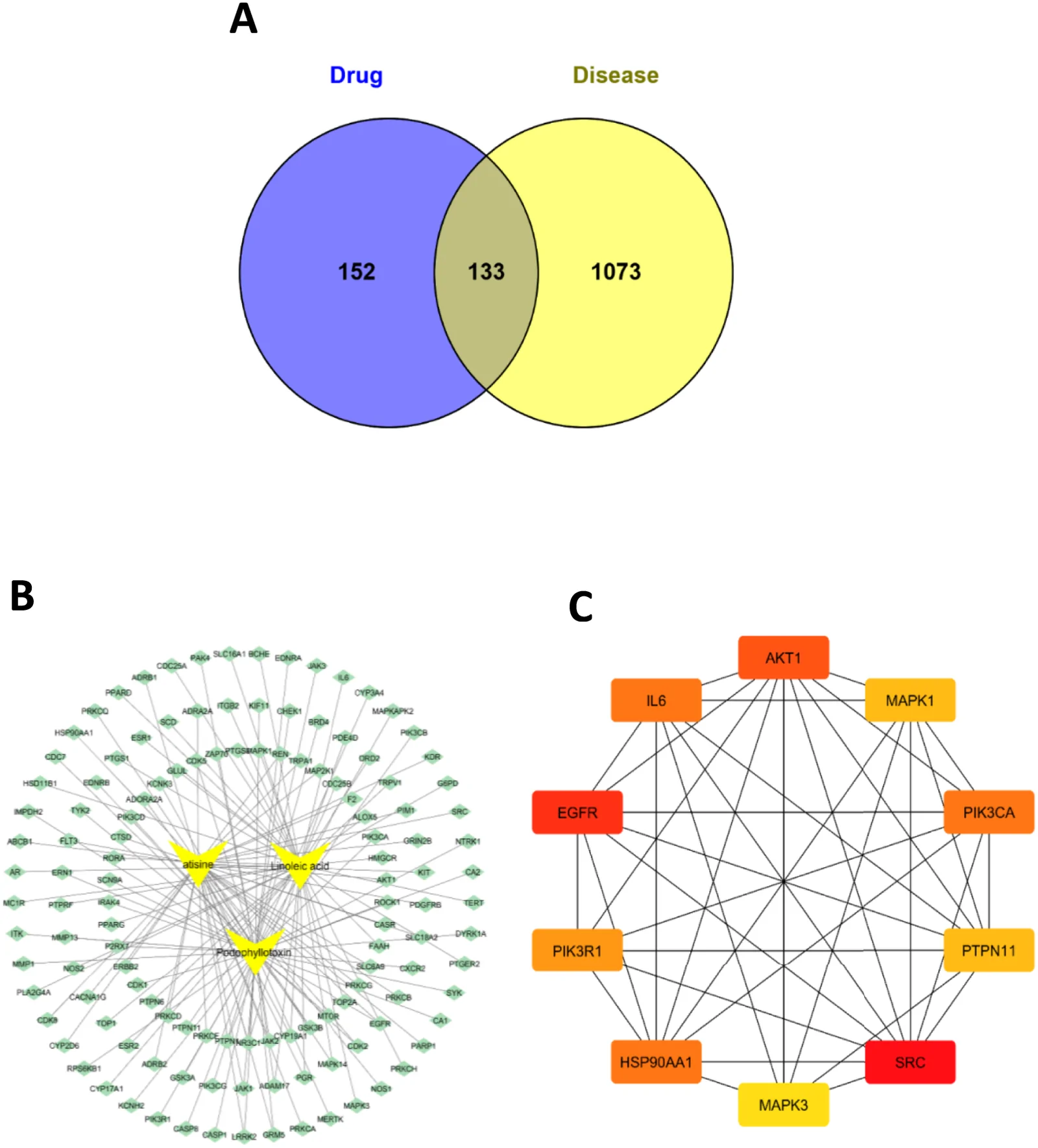
Analysis of network pharmacology of active compounds-cancer. **(A)** The Venn diagram of active compounds-cancer targets; **(B)** Topological network schematic of proteins targeted by active compounds and associated with cancer; **(C)** top 10 hub genes.

#### 4.7.2 Construction and analysis of the PPI network

By searching the public databases (Swiss target prediction, OMIM, Gene Cards), confining the result to “*H. sapiens*,” 327 targets related with compounds and 1,299 targets with cancer were collected, respectively. The utilization of Venny 2.0.2 facilitated the calculation of the intersection between compound targets and disease targets, resulting in 133 intersection targets. The resulting PPI network encompasses a total of 133 nodes and 565 edges, each representing a PPI. The average node degree is 8.5, with a local clustering coefficient of 0.497. The significance of each node within a complex network was estimated using three parameters: the degree, closeness, and centrality between the nodes in the network (Table 8). According to the topology properties of the network, there were ten targets in the network, corresponding to SRC, EGFR, AKT1, PIK3CA, HSP90AA1, IL6, PIK3R1, MAPK1, PTPN11, and MAPK3.

**TABLE 8:** Topological network schematic of proteins targeted by active compounds associated with cancer.

Name	Degree	Betweenness centrality	Closeness centrality
SRC	40	0.160836	0.543779
EGFR	37	0.084462	0.526786
AKT1	33	0.081995	0.524444
PIK3CA	31	0.022545	0.47012
HSP90AA1	31	0.107546	0.508621
IL6	31	0.149037	0.502128
PIK3R1	29	0.025365	0.464567
MAPK1	27	0.039119	0.491667
PTPN11	27	0.027225	0.453846
MAPK3	26	0.035968	0.483607

#### 4.7.3 GO and KEGG enrichment analyses

The examination of biological processes showed that target genes were notably enriched in activities like protein phosphorylation, response to chemical agents, and intracellular signal transmission. Regarding cellular components, enrichment was prominent in vesicles, cell junctions, and the integral part of the plasma membrane. The analysis of molecular functions highlighted the significance of protein serine/threonine/tyrosine kinase activity, kinase activity, and ATP binding. The KEGG pathway enrichment analysis revealed key pathways such as pathways in cancer, PI3K-Akt signaling pathway, and PD-L1 expression and PD-1 checkpoint pathway in cancer. A network analysis was developed to map the connections between ingredients, targets, and the top 10 pathways (Figure 11).

**FIGURE 11:**
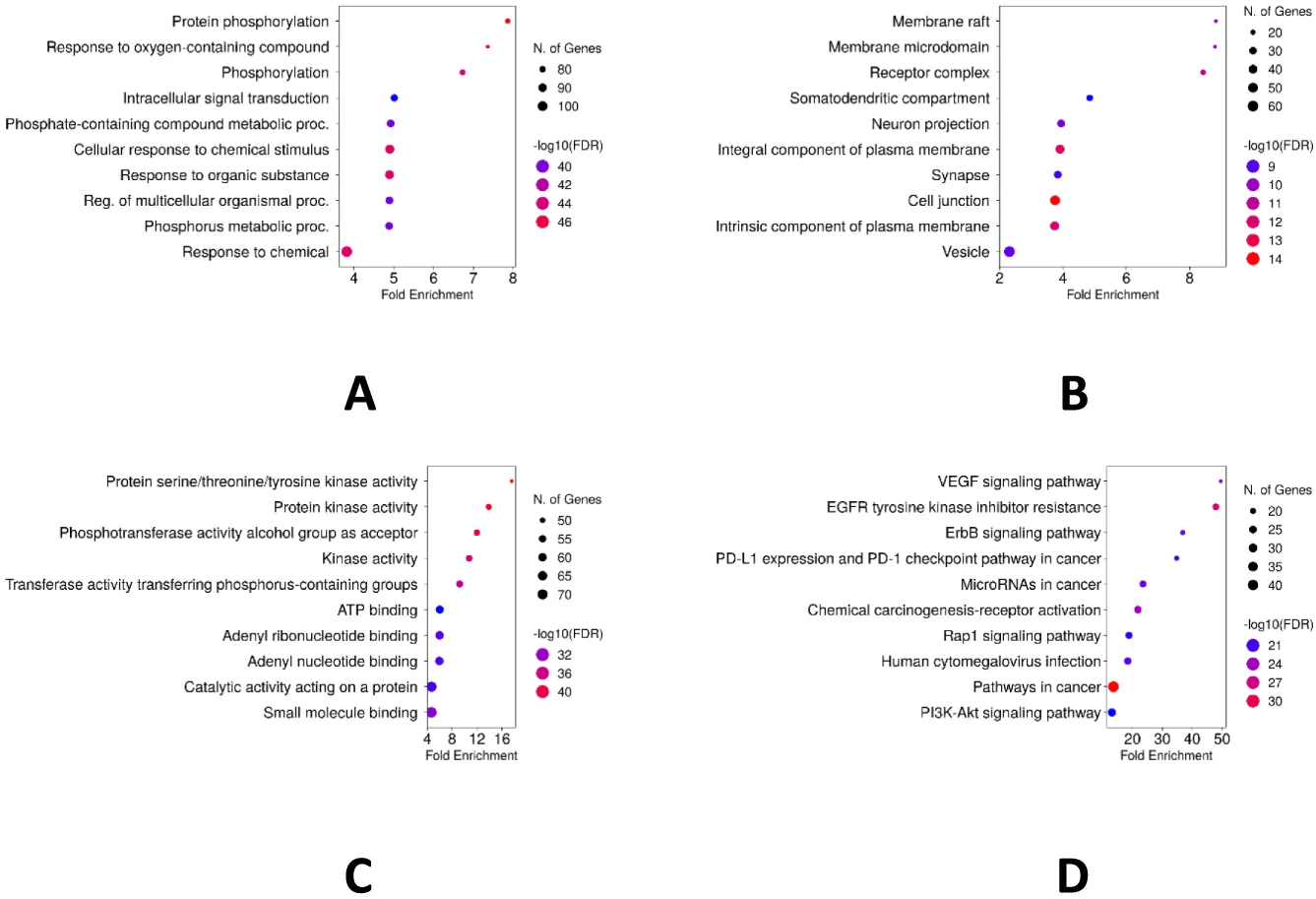
Enrichment analysis for gene ontology. **(A)** Biological process. **(B)** Molecular function. **(C)** Cellular component **(D)** KEGG Pathway.

## 5 Discussion

This study demonstrates that bioactive constituents from *A. heterophyllum* (atisine) and *P. hexandrum* (podophyllotoxin) exert potent anticancer effects against lung (A549) and prostate (LNCaP) cancer cells, supporting their potential development as modulators of inflammatory and androgen receptor-associated signaling pathways of inflammatory (IL-1R1) and hormonal (AR) signaling. Integration of *in vitro* cytotoxicity, ADMET profiling, molecular docking, molecular dynamics (MD) simulations, MM-GBSA and network pharmacology provides a coherent multiscale framework linking cellular phenotypes with molecular mechanisms. The strong activity and polypharmacology of podophyllotoxin align with extensive literature on podophyllotoxin-based natural products and derivatives as powerful cytotoxics and multitarget agents in oncology (Xie et al., 2025; Peña-Morán et al., 2016; Zi et al., 2019; Nguyen et al., 2022).

Podophyllotoxin displayed exceptional cytotoxicity (IC_50_ ≈ 0.04 µM) in both A549 and LNCaP cells, surpassing the reference drug doxorubicin (IC_50_ 0.22–0.25 µM). This is consistent with extensive evidence that podophyllotoxin and its derivatives (e.g., etoposide, teniposide) are among the most potent microtubule-destabilizing and/or topoisomerase II–targeting scaffolds, inducing G2/M arrest, DNA damage, and apoptosis across multiple tumor types, including lung and breast cancers (Peña-Morán et al., 2016; Nguyen et al., 2022; Choi et al., 2015; Rodríguez-López et al., 2021; Rai’dah et al., 2025). Several analogues exhibit sub-micromolar cytotoxicity in A549 other solid tumor models trigger robust apoptosis and cell-cycle arrest (Nguyen et al., 2025; Nguyen et al., 2022; Zi et al., 2019; Nguyen et al., 2024; Zi et al., 2018). The marked suppression of clonogenic growth (p < 0.001) further indicates that podophyllotoxin not only induces acute cytotoxicity but also abrogates long-term proliferative capacity, consistent with prior clonogenic data for aryltetralin lignans (Peña-Morán et al., 2016).

In contrast, atisine exhibited moderate but meaningful cytotoxicity (IC_50_ 2.1–2.3 µM) and significantly impaired colony formation (p < 0.05), suggesting lead-like antiproliferative behavior rather than weak adjunctive activity. Linoleic acid showed weak cytotoxicity (IC_50_ > 100 µM) and marginal clonogenic effects, consistent with its role as a bioactive lipid rather than a primary chemotherapeutic scaffold, as reported for lipid-modulating pathways in cancer and metastasis (Wen et al., 2019).

The differential activities of these compounds likely reflect their distinct chemotypes and target engagement profiles. Lignans such as podophyllotoxin possess rigid polycyclic frameworks favoring high-affinity interactions with cytoskeletal and nuclear proteins (Rodríguez-López et al., 2021; Rai’dah et al., 2025; Nguyen et al., 2022), whereas diterpenoid alkaloids like atisine may preferentially modulate signaling proteins and inflammatory pathways, as suggested for related diterpenoid-based anticancer scaffolds (Man et al., 2012). This divergence is supported by docking and MD analyses. Podophyllotoxin bound strongly to AR (−8.0 kcal/mol), forming key hydrophobic and hydrogen-bond interactions with GLU681, ARG752, TRP751, and THR755 within or near the ligand-binding domain critical for transcriptional activation. While most prior studies have focused on tubulin and topoisomerase II as primary targets (Rodríguez-López et al., 2021; Peña-Morán et al., 2016; Nguyen et al., 2022; Mishra et al., 2022; Choi et al., 2015), growing evidence indicates that podophyllotoxin derivatives can engage additional signaling proteins and kinases (Xie et al., 2025; Mishra et al., 2022; Lv et al., 2024), consistent with AR antagonism in prostate cancer cells.

Concurrently, podophyllotoxin demonstrated predicted binding interactions with IL-1R1 with high affinity (−7.6 kcal/mol), forming multiple hydrogen bonds with ARG9, GLU10, GLU11, and ASN30, suggesting interference with pro-inflammatory cytokine signaling involved in tumor promotion, angiogenesis, and immune evasion. This observation is conceptually aligned with reports that podophyllotoxin derivatives modulate immune-related pathways such as cGAS–STING (Xie et al., 2025).

Atisine showed its strongest binding to IL-1R1 (−8.1 kcal/mol), involving GLU25, PRO131, and VAL132, with favorable affinity toward AR (−7.2 kcal/mol). The tighter and more persistent IL-1R1–atisine interaction observed in MD simulations (RMSD <3 Å; MM-GBSA –71.25 to −49.77 kcal/mol) the computational analyses suggest that atisine may and immune-associated signaling pathways with secondary antiproliferative effects. This is consistent with growing recognition that plant-derived alkaloids and diterpenoids can exert anticancer activity through immune and inflammatory modulation rather than direct cytotoxicity alone (Zhou et al., 2020; Man et al., 2012). Given the established role of IL-1 family cytokines in cancer-associated inflammation, metastatic niche formation, and therapy resistance, targeting IL-1R1-mediated inflammatory signaling represents a promising complementary strategy (Dinarello, 2009).

ADMET and drug-likeness analyses further support the translational promise of atisine and podophyllotoxin. Both compounds comply with common oral drug design criteria and display favorable physicochemical properties. However, the predicted low human intestinal absorption (HIA) values observed for these compounds may limit their oral bioavailability, suggesting that formulation optimization, structural modification, or alternative delivery strategies may be required for future therapeutic development. Podophyllotoxin’s high plasma protein binding and tissue distribution are typical of lipophilic anticancer agents and consistent with ADME predictions for its derivatives (Nguyen et al., 2022; Zi et al., 2019; García et al., 2024). Although its clinical application has historically been limited by systemic toxicity and narrow therapeutic indices (Peña-Morán et al., 2016; Zi et al., 2019; Choi et al., 2015; Man et al., 2012), modern derivatization and formulation strategies—including glycosylation, targeted conjugation, and nanoformulations—have markedly improved safety and selectivity (Zi et al., 2019; Xie et al., 2025; Zi et al., 2018; García et al., 2024). Atisine showed moderate protein binding, better predicted bioavailability, and lower TPSA, consistent with trends for diterpenoid alkaloids as drug leads (Zhou et al., 2020), although its predicted P-gp substrate status suggests potential efflux-mediated resistance, a challenge shared by many plant-derived compounds (Xie et al., 2025; Lv et al., 2024).

Network pharmacology analysis further indicates that atisine and podophyllotoxin act through multitarget mechanisms. The identification of 133 shared targets and enrichment of core oncogenic hubs (SRC, EGFR, AKT1, PIK3CA, MAPK1/3, IL6) highlight simultaneous modulation of multiple cancer hallmarks. Enrichment of PI3K–Akt, MAPK, and immune-related pathways suggests interference with both tumor-intrinsic signaling and tumor–microenvironment crosstalk, supporting the advantage of polypharmacology in overcoming resistance in heterogeneous tumors such as lung and prostate cancers (Xie et al., 2025; Mishra et al., 2022; Lv et al., 2024; Man et al., 2012).

Taken together, these findings position atisine and podophyllotoxin as complementary leads within an integrated therapeutic framework. Podophyllotoxin, with nanomolar cytotoxicity and strong AR engagement, appears well suited as a direct cytotoxic and hormone-modulating agent, while atisine, with stable IL-1R1 binding and favorable ADMET characteristics, may function as an anti-inflammatory and signaling modulator that complements cytotoxic therapy. The limited efficacy of linoleic acid supports a potential adjuvant rather than standalone role.

### 5.1 Study limitations and future perspectives

The present study integrates experimental cytotoxicity assay, colony forming unit assay with computational analyses, including molecular docking, molecular dynamics simulations, ADMET prediction, MM-GBSA calculations, and network pharmacology. Although these analyses suggest potential modulation of IL-1R1-associated inflammatory signaling and androgen receptor pathways, direct experimental validation of these molecular targets through qRT-PCR, Western blotting, ELISA, RNA sequencing, or functional assays was not performed. Therefore, the proposed molecular mechanisms should be considered preliminary and hypothesis-generating. Future studies will focus on validating these predicted pathways using targeted molecular, cellular, and *in vivo* approaches.

## 6 Conclusion

In conclusion, this study demonstrates the promising anticancer potential of the natural compounds Atisine and Podophyllotoxin, isolated from *A. heterophyllum* and *P. hexandrum*, respectively, against lung and prostate cancer cell lines. The MTT and colony formation assays demonstrated dose-dependent cytotoxic and long-term antiproliferative effects, with Podophyllotoxin exhibiting greater potency than Atisine and Linoleic acid. Computational analyses, including ADMET profiling, molecular docking, molecular dynamics simulations, and network pharmacology, predicted favorable pharmacokinetic properties, stable interactions with the androgen receptor (AR) and type-I interleukin-1 receptor (IL-1R1), and the involvement of cancer-associated pathways such as the PI3K–Akt signaling pathway. However, these computational findings represent predicted molecular interactions and require experimental validation to confirm their biological relevance. Therefore, further mechanistic investigations using techniques such as qRT-PCR, Western blotting, ELISA, RNA sequencing, and *in vivo* studies are necessary to validate the predicted molecular targets and signaling pathways. Collectively, the present findings provide a strong preliminary foundation for the continued investigation and optimization of these natural compounds as potential candidates for anticancer drug development.

## Data Availability

The original contributions presented in the study are included in the article/Supplementary Material, further inquiries can be directed to the corresponding author.

## Glossary

ADMET: Absorption, Distribution, Metabolism, Excretion, and Toxicity
AR: Androgen Receptor
BBB: Blood–Brain Barrier
BP: Biological Process
CC: Cellular Component
DMEM: Dulbecco’s Modified Eagle Medium
DMSO: Dimethyl Sulfoxide
FBS: Fetal Bovine Serum
GO: Gene Ontology
HBA: Hydrogen Bond Acceptors
HBD: Hydrogen Bond Donors
**IC**_**50**_: Half-Maximal Inhibitory Concentration
IL-1R1: Interleukin-1 Receptor Type 1
KEGG: Kyoto Encyclopedia of Genes and Genomes
MD: Molecular Dynamics
MF: Molecular Function
MM-GBSA: Molecular Mechanics/Generalized Born Surface Area
MTT: 3-(4,5-Dimethylthiazol-2-yl)-2,5-Diphenyltetrazolium Bromide
nRot: Number of Rotatable Bonds
OMIM: Online Mendelian Inheritance in Man
P-gp: P-Glycoprotein
PBS: Phosphate-Buffered Saline
PDB: Protein Data Bank
PPB: Plasma Protein Binding
PPI: Protein–Protein Interaction
PTOX: Podophyllotoxin
QED: Quantitative Estimate of Drug-Likeness
RCSB: Research Collaboratory for Structural Bioinformatics
RMSD: Root Mean Square Deviation
RMSF: Root Mean Square Fluctuation
SD: Standard Deviation
SMILES: Simplified Molecular Input Line Entry System
TLC: Thin-Layer Chromatography
TPSA: Topological Polar Surface Area
Vdss: Volume of Distribution at Steady State
