# Supplementary material for: Targeting IL-1R1 and androgen receptor using natural compounds from Aconitum heterophyllum and Podophyllum hexandrum: an in silico and in vitro study
Source: Front Pharmacol. 2026 Oct 27;17:1909943. doi: 10.3389/fphar.2026.1909943 (PMC13748216; doi:10.3389/fphar.2026.1909943)
Supplement: Supplementary file 1 [file Supplementaryfile1.docx]

**NMR SPECTRAL ANALYSIS**

**Atisine**

1H NMR (400 MHz, DMSO) δ 11.90 (s, 1H), 5.34 – 5.23 (m, 6H), 3.54 (s, 3H), 2.71 (t, J = 6.0 Hz, 3H), 2.14 (t, J = 7.4 Hz, 6H), 1.99 (q, J = 6.5 Hz, 3H), 1.48 (t, J = 6.6 Hz, 23H), 1.31 (s, 4H). 13C NMR (150 MHz, DMSO) δ 174.67 (s), 151.91 (s), 146.58 (s), 146.43 (s), 136.52 (s), 136.43 (s), 135.00 (s), 130.71 (s), 128.27 (s), 109.06 (s), 108.41 (s), 106.31 (s), 100.96 (s), 71.01 (s), 70.55 (s), 59.88 (s), 55.85 (s), 44.07 (s), 43.34 (s), 40.14 (s), 40.03 (s), 39.90 (s), 39.77 (s), 39.63 (s), 39.50 (s), 39.36 (s), 39.22 (s), 39.08 (s).

**1H NMR**

**C^13^ NMR**

**Figure S2** 1HNMR and C13 NMR spectra of Atisine

**Podophylotoxin**

^1^H NMR (400 MHz, DMSO) δ 7.36 (s, 1H), 7.10 (s, 1H), 6.47 (s, 1H), 6.33 (s, 2H), 6.03 – 5.90 (m, 2H), 5.78 (d, *J* = 7.2 Hz, 1H), 4.62 (dd, *J* = 9.4, 7.5 Hz, 1H), 4.49 (d, *J* = 5.7 Hz, 2H), 4.09 (dd, *J* = 10.4, 8.6 Hz, 1H), 3.64 (s, 7H),
3.62 (s, 3H). ^13^C NMR (151 MHz, DMSO) δ 174.67 (s), 151.92 (s), 146.58 (s), 146.43 (s), 136.48 (d, *J* = 12.6 Hz), 135.00 (s), 130.71 (s), 128.27 (s), 109.06 (s), 108.41 (s), 106.31 (s), 100.96 (s), 71.01 (s), 70.55 (s), 59.88 (s), 55.85 (s), 44.06 (s), 43.34 (s).

**Figure S3** 1HNMR and C13 NMR spectra of Podophylotoxin

**Linoleic acid**

^1^H NMR (400 MHz, DMSO) δ 11.90 (s, 1H), 5.41 – 5.14 (m, 1H), 2.71 (t, *J* = 6.2 Hz, 1H), 2.14 (t, *J* = 7.4 Hz, 1H), 1.99 (q, *J* = 6.6 Hz, 1H), 1.55 – 1.42 (m, 1H), 1.26 (d, *J* = 13.8 Hz, 5), 0.84 (t, *J* = 6.8 Hz, 1H). ^13^C NMR (151 MHz, DMSO) δ 174.30 (s), 129.59 (s), 127.68 (s), 33.73 (s), 31.09 (s), 29 H.23 (d, *J* = 1.9 Hz), 28.87 (dd, *J* = 12.8, 8.7Hz), 26.75 (d, *J* = 5.0 Hz), 25.23 (s), 24.62 (s), 22.14 (s), 13.78 (s).

**Figure S4** 1HNMR and C13 NMR spectra of Linoleic acid

MD SIMULATION

**Figure S5** SSE for IL-1-atisine complex (a) Distribution (b) Timeline.

**Figure S6** SSE for IL-1-atisine complex (a) Distribution (b) Timeline.

**Figure S7** Protein-ligand interactions in A) AR-Podophyllotoxin complex and B) IL-1-atisine complex.

**Figure S8** Ligand Properties A) AR-Podophyllotoxin complex and B) IL-1-atisine complex.
